# The Hearsey Treatment of Blackwater Fever

**Published:** 1908-10-17

**Authors:** 


### SPECIAL ARTICLE.

### THE HEARSEY TREATMENT OF BLACKWATER FEVER.

Occasionally patients who have suffered from blackwater fever abroad, and who have returned to Great Britain, experience a relapse of this disease, and require treatment at the hands of a doctor who has never been abroad and who has not had an opportunity to treat a case before. The doctor will naturally refer to the text-book; it is surprising how little assistance he will get therefrom. The following brief account, therefore, of the treatment of blackwater fever may, perhaps, be of service. It is very highly spoken of in the journal of the Royal Army Medical Corps by Captain F. Hallam Hardy, R.A.M.C., who, during ten years, has acquired much experience upon the subject in Nyassaland.

Formerly the teaching was that quinine in large doses, champagne, and beef tea were to be administered. Then came a time when quinine was tabooed, and a variety of other remedies were successively recommended in its place. The treatment of blackwater fever became chaotic until, in 1899, definite rules were formulated by Dr. H. Hearsey. The treatment recommended by this officer has been attended by such an immense reduction in the mortality of the disease that the name blackwater fever has been robbed of many of its terrors—and this, from the mental point of view, in a severe case alone, means a great deal in coping with it.

The details of the Hearsey treatment are as follows: The patient is naturally in bed, and all precautions are taken against his getting any chill. As soon as possible after the onset of the illness a sufficient dose of calomel is given by the mouth; a quarter of a grain of morphia is injected hypodermically to allay restlessness and vomiting; the patient is wrapped in blankets; and hot-water bottles are put into the bed.

An hourly dose of a mixture containing half a drachm of liquor hydrargyri perchloridi and ten grains of sodium bicarbonate to a tablespoonful of water is given for the first twenty-four hours, and subsequently every two hours until the urine clears.

No food is given for the first twenty-four hours, unless there is a complete absence of vomiting, together with a strong inclination on the patient's part for something to eat. The kind of diet which is suitable in most cases consists of: (1) Small quantities of milk, with or without soda water; (2) Small quantities of freshly-made chicken broth; (3) Small quantities of white wine whey; (4) *Barley water ad libitum*, the patient being encouraged to drink it. Acid drinks, such as lemonade, champagne, or hock are absolutely prohibited. If towards the latter part of the illness alcohol seems requisite, brandy or whisky is the only form in which it should be exhibited. Beef extracts and essences are also strictly forbidden. No quinine is allowed. Digitalis may be indicated in some cases.

The great value of the above routine method of treatment is clearly demonstrated by the immediate decline in percentage mortality from blackwater fever following its use. Dr. Hearsey was able to report a series of twenty-one cases without a death. Almost as good results have been obtained by other practitioners. Whereas the average mortality used to be so great that one case in three proved fatal, the percentage of patients that die under the Hearsey treatment is very small.

There are a few minor details in the treatment that remain to be mentioned. Amongst others the value of saline solution in severe cases merits attention. It may be administered by intravenous or intracellular injection, but it serves its purpose equally well if it is given slowly and continuously per rectum. It is as well that the saline solution should contain not only sodium chloride but also sodium bicarbonate, the amount of the latter being as much as one or even two drachms per pint. It is of decided benefit in some cases.

Another point, and one upon which too much stress cannot be laid, is the importance of absolute recumbency during the illness and for at least seven days after it. Fatal syncope may result from getting out of bed to go to stool, either during the acute stage of the disease or during the first few days of convalescence.

Vomiting is sometimes a symptom of extreme urgency; it is relieved more effectually and speedily by very small injections of morphia than in any other way. Pains in the hepatic region are often a symptom of the disease, and sometimes they recur for months afterwards. They are best relieved by local warmth, especially by the application of hot stupes. Turpentine should not be employed in the latter, for although both turpentine and cantharides have at certain times been recommended in small doses as remedies for blackwater fever, there is no evidence that either does any good in the condition, and seeing that each can itself produce hæmaturia it is best to avoid them altogether.

